# Community norms for the eating disorder examination questionnaire (EDE-Q) among cisgender bisexual plus women and men

**DOI:** 10.1007/s40519-020-01070-8

**Published:** 2020-12-03

**Authors:** Jason M. Nagata, Emilio J. Compte, Stuart B. Murray, Rebecca Schauer, Erica Pak, Annesa Flentje, Matthew R. Capriotti, Micah E. Lubensky, Mitchell R. Lunn, Juno Obedin-Maliver

**Affiliations:** 1 Department of Pediatrics, University of California, San Francisco, 550 16th Street, 4th Floor, Box 0110, San Francisco, CA 94158 USA; 2 School of Psychology, Universidad Adolfo Ibáñez, Santiago, Chile; 3 Comenzar de Nuevo Treatment Center, Monterrey, México; 4 Department of Psychiatry and Behavioral Sciences, University of Southern California, Los Angeles, CA USA; 5 Department of Community Health Systems, University of California, San Francisco, San Francisco, CA USA; 6 Alliance Health Project, Department of Psychiatry and Behavioral Sciences, University of California, San Francisco, CA USA; 7 Department of Psychology, San José State University, San Jose, CA USA; 8 The PRIDE Study/PRIDEnet, Stanford University School of Medicine, Stanford, CA USA; 9 Division of Nephrology, Department of Medicine, Stanford University School of Medicine, Stanford, CA USA; 10 Department of Epidemiology and Population Health, Stanford University School of Medicine, Stanford, CA USA; 11 Department of Obstetrics and Gynecology, Stanford University School of Medicine, Stanford, CA USA

**Keywords:** Eating disorder, Bisexual, Cisgender, Norms, Sexual minorities

## Abstract

**Purpose:**

Cisgender bisexual plus (including bisexual, pansexual, and polysexual) women and men experience unique health concerns including eating disorders. The purpose of this study was to develop community norms for eating disorder attitudes and disordered eating behaviors in cisgender bisexual plus women and men using the Eating Disorders Examination Questionnaire (EDE-Q).

**Methods:**

Participants were cisgender bisexual plus women (*n* = 462) and men (*n* = 93) participants in The PRIDE Study, an existing study of sexual and gender minority people.

**Results:**

Mean and standard deviation of EDE-Q scores among cisgender bisexual plus women and men, respectively, were: Global (1.75 ± 1.26, 1.56 ± 1.18), Restraint (1.34 ± 1.44, 1.42 ± 1.53), Eating Concern (0.96 ± 1.13, 0.63 ± 0.96), Weight Concern 2.27 ± 1.55, 1.89 ± 1.46), and Shape Concern 42 ± 1.62, 2.30 ± 1.57). Among cisgender bisexual plus women and men, respectively, 27.5% and 22.6% scored in the clinically significant range on the Global score. Bisexual plus women and men reported any occurrence (≥ 1/28 days) of dietary restraint (19.3%, 23.7%), objective binge episodes (11.1%, 10.8%), excessive exercise (4.5%, 5.4%), self-induced vomiting (1.7%, 0.0%), and laxative misuse (0.4%, 1.1%), respectively. A lower percentage of age-matched cisgender bisexual plus women (18–25 years) reported any occurrence of objective binge episodes, self-induced vomiting, laxative misuse, and excessive exercise than previously published in young women. Age-matched cisgender bisexual plus men (18–26 years) reported higher weight concern subscale scores than previously published in young men.

**Conclusions:**

These norms should aid clinicians in applying and interpreting the EDE-Q scores of cisgender bisexual plus women and men.

**Level of evidence:**

Level V: cross-sectional descriptive study

## Introduction

Cisgender (i.e., those whose gender identity matches what is commonly associated with the sex assigned to them at birth) bisexual women and men experience unique health disparities in physical, mental, and sexual health outcomes [1]. In addition to bisexuality, other plurisexual identities (i.e.*,* romantic or sexual attraction and/ or behavior with members of more than one sex or gender)—such as pansexuality or polysexuality—are traditionally underrepresented in research [2]. In this paper, we refer to people who identify as bisexual, pansexual, or polysexual as “bisexual plus.” Bisexual plus people may experience prejudice, stigma, and discrimination based on sexual orientation from heterosexual communities, in response to which they may experience sexual minority stress and disordered eating [3–5]. While a growing literature identified disordered eating behaviors in sexual minority (i.e., non-heterosexual) populations, most investigations have grouped gay, lesbian, and bisexual plus populations together [6] and few focus exclusively on cisgender bisexual plus people.

There is little research on eating disorder norms for bisexual plus women [7]. One study found that bisexual women typically engage in more body checking (e.g., an individual’s tendency to repeatedly check their weight and shape through ritualistic weighing, compulsive mirror checking, and using the fit of clothes to judge weight changes) than heterosexual women [8]. Despite this group difference in body image observations, Henn et al. [8] reported no accordant evidence of a different likelihood or vulnerability to body image disturbances and eating disordered behaviors based on sexual orientation [8]. However, in another study, 10.6% of bisexual women reported both subjective binge eating and compensatory behavior compared to only 2.8% of lesbian women [9].

Research on eating disorder attitudes and behaviors in cisgender bisexual plus men is sparse [10, 11]. The existing research in presumably cisgender men suggests that bisexual men experience greater dissatisfaction with their bodies and a higher incidence of eating disorder symptoms compared to heterosexual men [12]. One study comparing rates of disordered eating behaviors among university men found the highest rates of objective binge eating among men who have sex with either men or women (29%) compared to men who have sex with men (6%) or men who have sex with women (10%) [13]. Among bisexual men, exercise frequency has been found to be significantly associated with eating disorder symptomology, suggesting that, when compared with any other subgroup, bisexual men are more likely to use exercise as a method of weight control [14].

Although eating disorder attitudes and behaviors have been noted to be higher in presumably cisgender and heterosexual women than men [15, 16], gender differences are unknown among cisgender bisexual plus women and men. Given higher eating disorder symptoms in bisexual compared to heterosexual men [12], gender differences among bisexual plus women and men may be attenuated.

This study aims to (1) develop community norms for eating disorder attitudes and disordered eating behaviors using the EDE-Q among cisgender bisexual plus women and men, (2) compare community norms of the EDE-Q among cisgender bisexual plus women (age-matched) with those previously published in presumed cisgender heterosexual young women [16], and (3) compare community norms of the EDE-Q among cisgender bisexual plus men (age-matched) with those previously published in presumed cisgender heterosexual young men [15]. We hypothesized that EDE-Q norms would be higher in cisgender bisexual plus women and men than those previously published in presumed cisgender heterosexual young women [16] and men [15], respectively.

## Methods

### Study design and population

This study uses cross-sectional data from The Population Research in Identity and Disparities for Equality (PRIDE) Study, a national longitudinal cohort study of sexual and gender minority (SGM) adults in the US which include, but are not limited to, people who identify as lesbian, gay, bisexual, transgender, and/or queer. This ancillary study invited all The PRIDE Study participants to participate in a cross-sectional, self-reported, online survey from April to August 2018. The PRIDE Study engages, recruits, and consents participants. It also collects data on a web-based platform that is accessible from any mobile device or computer with Internet access (www.pridestudy.org). Associated with The PRIDE Study is PRIDEnet, a national network of organizations and individuals created to actively engage SGM communities in research for and beyond The PRIDE Study. Participants in The PRIDE Study are recruited through PRIDEnet constituents; social media advertising; digital communications (blog posts, newsletters); distribution of The PRIDE Study-branded promotional items; direct outreach at conferences, symposiums, and events; and word-of-mouth. Inclusion criteria included age ≥ 18 years, self-identification as a SGM person, residing in the US or its territories, and written and oral proficiency in English to read and answer questions. Further description of the cohort population, demographics, and technology that supports the study have been previously described [17, 18]. This study was approved by the University of California, San Francisco Institutional Review Board on 2 February 2018 (#16-21213), as well as The PRIDE Study’s Research Advisory and Participant Advisory Committees.

We included cisgender bisexual plus women and men in this analysis. Participants were asked about their gender identity (“What is your current gender identity?”) and the sex assigned to them at birth (“What sex were you assigned at birth on your original birth certificate?”). Cisgender women were defined as persons who responded “woman” for gender identity and “female” for sex assigned at birth. Cisgender men were defined as persons who responded “man” for gender identity and “male” for sex assigned at birth. Participants who indicated sexual orientation as bisexual (*n* = 388 women, 82 men), pansexual (*n* = 221 women, 32 men), or polysexual (*n* = 3 women, 0 men, write-in) were included as bisexual plus. Participants could indicate more than one sexual orientation. Participants who reported multiple gender identities or sexual orientations other than bisexual, pansexual, or polysexual (such as gay, lesbian, or queer) were excluded. Of the 4285 participants from The PRIDE Study who completed the questionnaire, 507 were cisgender bisexual plus women and 100 were cisgender bisexual plus men. Participants who fully completed all of the EDE-Q questions (462 cisgender bisexual plus women and 93 cisgender bisexual plus men) were included in the current study. In addition, data from 978 cisgender gay men (*M* age = 42.0, SD = 15.1, range 18 to 82 years) and 476 cisgender lesbian women from previously published reports of The PRIDE Study [19, 20] were considered for group comparisons across genders. For the purpose of this study, only participants with no missing values in the EDE-Q were considered.

### Measures

The EDE-Q is a self-report questionnaire that assesses disordered eating attitudes and behaviors over the previous 28 days [21]. The measure provides four attitudinal subscale scores: Restraint (5 items), Eating Concern (5 items), Shape Concern (8 items), and Weight Concern (5 items). An overall Global score is the mean of the four subscale scores. Responses are on a 7-point ordinal response; higher scores reflect greater eating-related concerns or behaviors. Frequencies of disordered eating behaviors (e.g., binge eating, compensatory behaviors) are assessed. Cronbach’s alpha in this study for cisgender bisexual plus women and men, respectively, were as follows: Global score (0.94 and 0.93), Restraint (0.79 and 0.84), Eating Concern (0.82 and 0.81), Weight Concern (0.84 and 0.83), and Shape Concern (0.91 and 0.89). A cutoff of > 2.48 as a marker of clinical significance (range 0–6; higher scores indicate greater symptoms) on the Global score was utilized based on a cut point with maximum sensitivity and specificity as determined by receiver operator characteristics (ROC) curve analysis for the 28-item EDE-Q established by Machado et al. (2020) [22].

In the EDE-Q, the frequency of binge eating and compensatory behaviors were assessed by the number of episodes occurring during the past 28 days, in accordance with previous literature [15, 16, 19, 20, 23–27] to allow comparisons across studies. Any occurrence was defined as ≥ 1 episode in the past 28 days [15, 16]. Regular occurrences of dietary restraint were defined as going for long periods of time (≥ 8 h) without eating anything to influence shape or weight for ≥ 13 days over the past 28 days as previously defined [15, 16, 28]. The choice of the cut-off for dietary restraint corresponded to an average of > 3 days per week. Thus, the cutoff was based on ≥ 3 on the 0–6 scale of the EDE-Q item 2. A rating of 4 on items/subscales of the EDE-Q has been used as a cut-off for clinical severity [15, 16], so the selection of the cut-off corresponding to a rating of ≥ 3 allowed for inclusion of a somewhat lower frequency given that the fasting behavior represented an extreme form of dietary restraint [15, 28]. The cutoff is also consistent with the clinical cutoff of > 2 identified by Machado et al. [22]. Regular occurrences of excessive exercise were defined as exercising in a driven or compulsive way as a means of controlling weight, shape or amount of fat, or burning off calories for ≥ 20 days over the past 28 days [15, 16]. The choice of the cutoff for driven or compulsive exercise was based on an average of ≥ 5 days per week, and this higher threshold was selected to enhance the likelihood that the cut-off reflects clinical severity, given that the item may not clearly distinguish between pathological and adaptive forms of exercise [15, 16]. For all other behaviors (objective binge episodes, self-induced vomiting, and laxative misuse), regular occurrence was defined as ≥ 4 occurrences over the past 28 days [15, 16], which would average to at least one episode per week and is consistent with the current Diagnostic and Statistical Manual, 5th Edition (DSM-5) criteria for bulimia nervosa and binge eating disorder [15, 16, 29].

Demographic information (age, race, ethnicity, and education), weight, and height were based on self-report. The standard formula weight (kilograms) divided by height (meters) squared was used to determine body mass index (BMI = weight/height^2^). Participants were also asked: “Has a mental health professional or physician ever told you that you have an eating disorder such as anorexia nervosa, bulimia nervosa, or binge eating disorder?” If affirmative, participants were asked to specify which type (participants could select more than one option). Options included anorexia nervosa, bulimia nervosa, binge eating disorder, or other/not specified.

### Data analysis

SPSS 20.0 was used for all analyses. Data are presented as mean (SD), median (IQR), and percentage. Associations between participant’s BMI and EDE-Q subscales and global scores were assessed through Pearson product-moment correlations coefficient; values of *r* > 0.10 were considered weak, *r* > 0.30 were considered moderate, and *r* > 0.50 were considered strong correlations [30]. The Student-*t* test was used for group comparisons across EDE-Q subscales and Global score, and *Z*‐tests (or Fisher’s exact tests) were conducted for comparisons across the proportions of individuals who reported each ED behavior. We assessed group comparisons of norms in the present sample of cisgender bisexual plus women to norms for cisgender lesbian women previously published from The PRIDE Study [20]. We also assessed group comparisons of norms in the present sample of cisgender bisexual plus men to norms for cisgender gay men previously published from The PRIDE Study [19]. In addition, we selected previously published norms of presumed cisgender heterosexual women [16] and men [15] as comparison groups as they were samples that most closely matched The PRIDE Study’s samples (e.g., US-based, non-clinical, community samples of adults). These samples were presumed to be predominantly cisgender and heterosexual as comprehensive gender identity and sexual orientation were not assessed [15, 16]. These previously published samples were of young adults; thus, we analyzed a subset of age-matched cisgender bisexual plus individuals for comparison. We calculated norms in a subset (*N* = 144) of cisgender bisexual plus women from The PRIDE Study (ages 18–25 years) to compare norms with those previously published in presumed cisgender heterosexual young adult women [16]. We calculated norms in a subset (*N* = 17) of cisgender bisexual plus men from The PRIDE Study (ages 18–26 years) to compare norms with those previously published in presumed cisgender heterosexual young adult men [15]. Finally, given the significant gender differences that were previously reported in norms of presumed cisgender heterosexual undergraduate women and men [15, 16], we assessed for gender differences in the norms among age-matched participants in our samples of cisgender bisexual plus men and women. Two‐tailed tests with an adjusted *p*‐value (Bonferroni) were set at 0.005 for significance.

### Power calculations

Given a sample size of presumed cisgender heterosexual women (*n* = 723) and cisgender bisexual plus women (*n* = 144) [16], and using an estimated mean for EDE-Q Global Score (1.74 ± 1.30), our study had statistical power (alpha = 0.05, two-sided) of > 80% to detect a 0.33 difference in EDE-Q Global Score. Given a sample size of presumed cisgender heterosexual men (*n* = 404) and cisgender bisexual plus men (*n* = 17) [15], and using an estimated mean for EDE-Q Global Score (1.09 ± 1.00), our study had statistical power (alpha = 0.05, two-sided) of > 80% to detect a 0.61 difference in EDE-Q Global Score.

## Results

### Cisgender bisexual plus women

A total of 462 cisgender bisexual plus women participated in this study (Table 1). The mean age was 31.9 years (SD = 9.4) ranging from 18 to 71 years of age. The mean BMI was 28.8 kg/m^2^ (SD = 8.2). A total of 82.0% of the participants identified as White (non-Hispanic/Latino), 4.8% as Hispanic/Latino, 3.0% as Asian or Pacific Islander (non-Hispanic/Latino), 2.6% as Black (non-Hispanic/Latino), 0.2% as Native American, and 7.6% as other or multiracial. In addition, 79.6% of participants had completed a college degree or higher. Overall, 11.3% of participants reported having ever been told by a mental health provider or physician that they had an eating disorder, including anorexia nervosa (5.4%), bulimia nervosa (3.2%), binge eating disorder (3.7%), or another or unspecified eating disorder (3.4%).

**Table 1:** Demographic characteristics of cisgender bisexual plus women (*N* = 462) and men (*N* = 93) from The PRIDE Study

	Cisgender bisexual plus women	Cisgender bisexual plus men
*N*	462	93
Demographic characteristics	Mean ± SD/%	Mean ± SD/%
Age, years	31.9 ± 9.4	38.3 ± 12.8
Race/ethnicity		
White	82.0%	79.6%
Hispanic/Latino	4.8%	2.2%
Asian/Pacific Islander	3.0%	7.5%
Black/African American	2.6%	2.2%
Native American	0.2%	0.0%
Multiracial/Other	7.6%	8.5%
Educational attainment		
College degree or higher	79.6%	80.2%
Body mass index (BMI), kg/m^2^	28.8 ± 8.2	28.1 ± 7.2

Mean scores, standard deviations, and percentile ranks for the EDE-Q subscales and Global Score are presented in Table 2. Among cisgender bisexual plus women, 5.9% scored in the clinically significant range on the Restraint, 3.9% on the Eating Concern, 19.0% on the Weight Concern, 19.7% on the Shape Concern subscales, and 5.8% on the Global score scale. In addition, 27.5% of cisgender bisexual plus women scored above the 2.48 cutoff score suggested by Machado et al. (2020) [22].

**Table 2:** Distribution of means, standard deviations, and percentile ranks for Eating Disorder Examination Questionnaire (EDE-Q) Global and subscale scores among cisgender bisexual plus women (*N* = 462) and men (*N* = 93) from the PRIDE Study

	Cisgender bisexual plus women (*N* = 462)					Cisgender bisexual plus men (*N* = 93)				
	EDE-Q Restraint	EDE-Q Eating Concern	EDE-Q Weight Concern	EDE-Q Shape Concern	EDE-Q Global	EDE-Q Restraint	EDE-Q Eating Concern	EDE-Q Weight Concern	EDE-Q Shape Concern	EDE-Q Global
Median (standard deviation)	1.34 (1.44)	0.96 (1.13)	2.27 (1.55)	2.42 (1.62)	1.75 (1.26)	1.42 (1.53)	0.63 (0.96)	1.89 (1.46)	2.30 (1.57)	1.56 (1.18)
Range	0–6.00	0–5.20	0–6.00	0–6.00	0–5.50	0–6.00	0–4.40	0–6.00	0–6.00	0–5.35
Percentile rank										
5	0	0	0	0.25	0.18	0	0	0	0.25	0.16
10	0	0	0.40	0.50	0.36	0	0	0.20	0.38	0.28
15	0	0	0.60	0.75	0.45	0	0	0.40	0.51	0.33
20	0	0	0.80	0.88	0.59	0	0	0.60	0.75	0.43
25	0	0.20	1.00	1.13	0.72	0	0	0.80	1.06	0.64
30	0.20	0.20	1.20	1.25	0.84	0.20	0	0.84	1.38	0.78
35	0.40	0.20	1.40	1.38	1.01	0.20	0	1.00	1.50	0.92
40	0.40	0.40	1.60	1.63	1.17	0.40	0.20	1.20	1.70	1.04
45	0.60	0.40	2.00	1.88	1.31	0.60	0.20	1.40	1.88	1.17
50	0.80	0.40	2.00	2.25	1.49	1.00	0.20	1.60	2.00	1.26
55	1.20	0.60	2.40	2.50	1.63	1.20	0.20	1.74	2.21	1.44
60	1.20	0.80	2.60	2.75	1.80	1.80	0.40	2.08	2.35	1.62
65	1.60	1.00	2.80	300	2.09	1.82	0.40	2.40	2.63	1.78
70	1.80	1.20	3.20	3.25	2.35	2.20	0.76	2.56	2.98	2.07
75	2.40	1.40	3.40	3.66	2.62	2.40	0.80	2.80	3.38	2.25
80	2.60	1.80	3.80	3.88	2.94	2.44	1.00	3.04	3.90	2.58
85	3.00	2.00	4.20	4.38	3.20	3.00	1.40	3.60	4.50	2.83
90	3.60	2.74	4.60	4.88	3.59	3.60	2.12	4.40	4.88	3.32
95	4.17	3.40	5.00	5.38	4.11	4.86	2.86	4.66	5.24	3.78
99	5.55	4.55	5.87	6.00	5.26	–	–	–	–	–

BMI was significantly positively correlated with all the subscales and the Global score of the EDE-Q. Significant weak correlations were observed between participants’ BMI and the Eating Concern (*r* = 0.22, *p* < 0.001), and Shape Concern (*r* = 0.29, *p* < 0.001) subscales, and the Global score (*r* = 0.28, *p* < 0.001). A moderate correlation was observed between BMI and the Weight Concern (*r* = 0.36, *p* < 0.001). No significant correlation was observed between participants’ BMI and the Restraint subscale (*r* = 0.07, *p* = 0.141).

Any occurrence and regular occurrences of key disordered eating behaviors are presented in Table 3. At least one episode of dietary restraint during the previous 28 days was observed for almost 20% of cisgender bisexual plus women. Of the total sample, more than 10% of the participants endorsed at least one episode of objective binge eating, and almost 5% reported at least one episode of excessive exercise over the past 28 days. Episodes of self-induced vomiting (1.7%) and laxative misuse (0.4%) in the last 28 days were rarely observed.

**Table 3:** Proportion of cisgender bisexual plus women and men engaging in disordered eating behaviors in The PRIDE Study

	Cisgender bisexual plus women (*N* = 462)		Cisgender bisexual plus men (*N* = 93)	
Disordered eating behavior	Any occurrence (%)	Regular occurrence (%)	Any occurrence (%)	Regular occurrence (%)
Dietary restraint	19.3	5.6	23.7	8.6
Objective binge episodes	11.1	3.7	10.8	6.5
Self-induced vomiting	1.7	0.9	0.0	0.0
Laxative misuse	0.4	0.4	1.1	0.0
Excessive exercise	4.5	0.4	5.4	2.2

Regular occurrence of dietary restraint was defined as going for long periods of time (8 h) without eating anything to influence shape or weight for ≥ 13 days over the past 28 days. Regular occurrence of excessive exercise was defined as exercising in a driven or compulsive way as a means of controlling weight, shape or amount of fat, or burning off calories for ≥ 20 days over the past 28 days. For all other behaviors, regular occurrence was defined as ≥ 4 occurrences over the past 28 days

Table 4 shows differences in the EDE-Q subscales, the EDE-Q Global score, and disordered eating behaviors between cisgender bisexual plus women and cisgender lesbian women. No significant differences were observed in either attitudinal subscales or disordered eating behaviors in these two groups.

**Table 4:** Comparisons of eating attitudes and disordered eating behaviors in cisgender bisexual plus women (*N* = 462) to cisgender lesbian women (*N* = 476), and cisgender bisexual plus men (*N* = 93) to cisgender gay men (*N* = 978) from The PRIDE Study

	Cisgender bisexual plus women from The PRIDE Study	Cisgender lesbian women from The PRIDE Study	*t*-test	*p*	Cisgender bisexual plus men from The PRIDE Study	Cisgender gay men from The PRIDE Study	*t*-test	*p*
Eating attitudes	Mean (standard deviation)				Mean (standard deviation)			
EDE-Q Restraint	1.34 (1.44)	1.40 (1.41)	− 0.67	0.506	1.42 (1.53)	1.54 (1.43)	− 0.73	0.469
EDE-Q EC	0.96 (1.13)	0.80 (1.05)	2.20	0.028	0.63 (0.96)	0.63 (0.98)	0.02	0.987
EDE-Q WC	2.27 (1.55)	2.06 (1.48)	2.09	0.037	1.89 (1.46)	1.91 (1.47)	− 0.12	0.902
EDE-Q SC	2.42 (1.62)	2.22 (1.57)	1.94	0.053	2.30 (1.57)	2.41 (1.62)	− 0.60	0.550
EDE-Q Global	1.75 (1.26)	1.62 (1.19)	1.58	0.115	1.56 (1.18)	1.62 (1.17)	− 0.47	0.642

	Cisgender bisexual plus women from The PRIDE Study	Cisgender lesbian women from The PRIDE Study	*Z*-test	*p*	Cisgender bisexual plus men from The PRIDE Study	Cisgender gay men from The PRIDE Study	*Z*-test	*p*
Disordered eating behaviors	Any occurrence (%)				Any occurrence (%)			
Dietary restraint	19.3	14.7	1.86	0.062	23.7	19.8	0.88	0.380
Objective binge episodes	11.1	10.1	0.48	0.634	10.8	10.9	0.06	0.956
Self-induced vomiting	1.7	0.4	–	0.062^†^	–	0.6	–	–
Laxative misuse	0.4	0.2	–	0.620^†^	1.1	1.1	–	0.999^†^
Excessive exercise	4.5	5.9	0.92	0.358	5.4	10.1	1.48	0.140

*EDE-Q* Eating Disorder Examination-Questionnaire, *EDE-Q EC* Eating Concern subscale, *EDE-Q WC* Weight Concern subscale, *EDE-Q SC* Shape Concern subscale, *EDE-Q Global* Global score

Any occurrence was defined as ≥ 1 episode in the past 28 days. EDE-Q scores were compared using independent samples *t*-tests. Proportions of disordered eating behaviors were compared with *Z*-tests or Fisher’s exact tests

^†^Fisher’s exact test

*p < .005 (after Bonferroni correction)

Table 5 shows group comparison in EDE-Q subscales, Global score, and disordered eating behaviors between age-matched cisgender bisexual plus women from The PRIDE Study with presumed cisgender heterosexual women from previously published norms [16]. No significant differences were observed between cisgender bisexual plus women and cisgender women in the attitudinal subscales or the Global score of the EDE-Q. However, a higher percentage of presumed cisgender heterosexual women reported any occurrence of objective binge episodes, self-induced vomiting, laxative misuse, and excessive exercise than cisgender bisexual plus women. No significant differences were observed in occurrence of dietary restraint between cisgender bisexual plus women and cisgender women [16].

**Table 5:** Comparisons of eating attitudes and disordered eating behaviors in a subsample of bisexual plus women 18–25 years old (*n* = 144) and bisexual plus men 18–26 years old (*n* = 17) in The PRIDE Study to age-matched cisgender heterosexual^ women from Luce et al. [16] sample (*N* = 723) and cisgender heterosexual^ men from the Lavender et al. [15] sample (*N* = 404)

	Cisgender bisexual plus women from The PRIDE Study	Cisgender heterosexual^ women from Luce et al. [16]	*t*-test	*p*	Cisgender bisexual plus men from The PRIDE Study	Cisgender heterosexual^ men from Lavender et al. [15]	*t*-test	*p*
Eating attitudes	Mean (standard deviation)				Mean (standard deviation)			
EDE-Q Restraint	1.30 (1.42)	1.62 (1.54)	− 2.31	0.021	1.42 (1.56)	1.04 (1.19)	1.27	0.205
EDE-Q EC	1.00 (1.13)	1.11 (1.11)	− 1.08	0.099	0.88 (1.16)	0.43 (0.77)	2.31	0.021
EDE-Q WC	2.37 (1.58)	1.97 (1.56)	2.80	0.005	2.26 (1.54)	1.29 (1.27)	3.06	0.002*
EDE-Q SC	2.47 (1.53)	2.27 (1.54)	1.42	0.156	2.44 (1.45)	1.59 (1.38)	2.48	0.014
EDE-Q Global	1.79 (1.25)	1.74 (1.30)	0.42	0.675	1.74 (1.27)	1.09 (1.00)	2.60	0.009

	Cisgender bisexual plus women from The PRIDE Study	Cisgender heterosexual^ women from Luce et al. [16]	*Z*-test	*p*	Cisgender bisexual plus men from The PRIDE Study	Cisgender heterosexual^ men from Lavender et al. [15]	*Z*-test	*p*
Disordered eating behaviors	Any occurrence (%)				Any occurrence (%)			
Dietary restraint	25.0	25.9	0.22	0.828	29.4	24.0	–	0.783^†^
Objective binge episodes	8.3	21.3	3.61	< .001*	11.8	25.0	–	0.214^†^
Self-induced vomiting	1.4	8.8	3.08	0.002*	–	3.2	–	–
Laxative misuse	0.7	8.3	3.26	0.001*	–	2.7	–	–
Excessive exercise	4.9	30.8	6.45	< .001*	11.8	31.4	1.72	0.085

*EDE-Q* Eating Disorder Examination-Questionnaire, *EDE-Q EC* Eating Concern subscale, *EDE-Q WC* Weight Concern subscale, *EDE-Q SC* Shape Concern subscale, *EDE-Q Global* Global score

Any occurrence was defined as ≥ 1 episode in the past 28 days. EDE-Q scores were compared using independent samples *t*-tests. Proportions of disordered eating behaviors were compared with *Z*-tests or Fisher’s exact tests

^Predominantly cisgender and heterosexual are presumed as comprehensive gender identity and sexual orientation assessment were not performed in Luce et al. [16] or Lavender et al. [15]

^†^Fisher’s exact test

^*^p < .005 (after Bonferroni correction)

### Cisgender bisexual plus men

A total of 93 cisgender bisexual plus men participated in this study (Table 1). The mean age was 38.3 years old (SD = 12.8) in a range of 20 to 76 years of age. The mean BMI was 28.1 kg/m^2^ (SD = 7.2). A total of 79.6% of the participants identified as White (non-Hispanic/Latino), 7.5% as Asian or Pacific Islander (non-Hispanic/Latino), 2.2% as Black (non-Hispanic/Latino), 2.2% as Hispanic/Latino, and 8.5% as other or Multiracial. In addition, 80.2% of participants had completed a college degree or higher. Overall, 3.2% of participants reported being told by a mental health provider or physician that they had an eating disorder, including anorexia nervosa (3.2%), bulimia nervosa (1.1%), or other/not specified (1.1%).

Mean scores, standard deviations, and percentile ranks for the EDE-Q subscales and Global score are presented in Table 2. Among cisgender bisexual plus men, 7.5% scored in the clinically significant range on the Restraint, 2.2% on the Eating Concern, 12.9% on the Weight Concern, 19.4% on the Shape Concern subscales, and 2.2% on the Global score scale. In addition, 22.6% of cisgender bisexual plus men scored above the 2.48 cutoff score suggested by Machado et al. (2020) [22].

BMI was moderately positively correlated with the Eating Concern (*r* = 0.39, *p* < 0.001) and Shape Concern (*r* = 0.40, *p* < 0.001) subscales, as well as the Global score (*r* = 0.44, *p* < 0.001). Also, BMI was strongly positively correlated with the Weight Concern (*r* = 0.52, *p* < 0.001). No significant correlation was observed between participants’ BMI and the Restraint subscale (*r* = 0.19, *p* = 0.073).

Any occurrence and regular occurrences of disordered eating behaviors are presented in Table 3. Nearly one quarter of participants reported any dietary restraint during the previous 28 days. Close to 10% of the participants endorsed at least one episode of objective binge eating and around 5% reported at least one episode of excessive exercise over the past 28 days. Episodes of self-induced vomiting were not observed, and any laxative misuse in the last 28 days was reported in 1.1% of the cases (*n* = 1).

Table 4 shows comparisons in the EDE-Q subscales, the EDE-Q Global score, and disordered eating behaviors between cisgender bisexual plus men and cisgender gay men. No differences were observed in either attitudinal subscales or key ED behaviors across groups.

Table 5 shows group comparison in EDE-Q subscales, Global score, and disordered eating behaviors between age-matched cisgender bisexual plus men from The PRIDE Study with presumed cisgender heterosexual men from previously published norms [15]. Cisgender bisexual plus men had a higher Weight Concern subscale score (2.26) than presumed cisgender heterosexual men previously published (1.29). Attitudinal scores in cisgender bisexual plus men compared to presumed cisgender heterosexual men and cisgender men included Eating Concern (0.88 vs 0.43, *p* = 0.021), Shape Concern (2.44 vs 1.59, *p* = 0.014), and Global score (1.74 vs 1.09, *p* = 0.009); however, these did not reach the *p* < 0.005 threshold after the Bonferroni correction. In terms of disordered eating behaviors, no significant differences were observed for dietary restraint, objective binge episodes, and excessive exercise. Contrary to presumed cisgender heterosexual men, cisgender bisexual plus men did not report any occurrence of self-induced vomiting and laxative misuse in the past 28 days.

Table 6 shows group comparisons in EDE-Q subscales, the EDE-Q Global score, and key ED behaviors between cisgender bisexual plus women and men. With the exception of the Eating Concern subscale, where cisgender bisexual plus women showed higher levels than cisgender bisexual plus men, no differences were observed across attitudinal and behavioral items of the EDE-Q between cisgender bisexual plus women and men.

**Table 6:** Comparisons of eating attitudes and disordered eating behaviors in cisgender bisexual plus women (*N* = 462) and cisgender bisexual plus men (*N* = 93) from The PRIDE Study

Eating attitudes	Cisgender bisexual plus women	Cisgender bisexual plus men	*t*-test	*p*
	M (SD)	M (SD)		
EDE-Q Restraint	1.34 (1.44)	1.42 (1.53)	− 0.51	0.611
EDE-Q EC	0.96 (1.13)	0.63 (0.96)	2.64	0.008
EDE-Q WC	2.27 (1.55)	1.89 (1.46)	2.18	0.030
EDE-Q SC	2.42 (1.62)	2.30 (1.57)	0.67	0.504
EDE-Q Global	1.75 (1.26)	1.56 (1.18)	1.33	0.185

Disordered eating behaviors	Cisgender bisexual plus women	Cisgender bisexual plus men	*Z*-test	*p*
	Any occurrence (%)	Any occurrence (%)		
Dietary restraint	19.3	23.7	0.97	0.334
Objective binge episodes	11.1	10.8	0.08	0.936
Self-induced vomiting	1.7	–	–	–
Laxative misuse	0.4	1.1	–	0.426^†^
Excessive exercise	4.5	5.4	–	0.788^†^

*M* mean, *SD* standard deviation, *EDE-Q* Eating Disorder Examination-Questionnaire, *EDE-Q EC* Eating Concern subscale, *EDE-Q WC* Weight Concern subscale, *EDE-Q SC* Shape Concern subscale, *EDE-Q Global* Global score

Any occurrence was defined as ≥ 1 episode in the past 28 days. EDE-Q scores were compared using independent samples *t*-tests. Proportions of disordered eating behaviors were compared with *Z*-tests or Fisher’s exact tests

^†^Fisher’s exact test

*p < .005 (after Bonferroni correction)

## Discussion

We describe community norms for the EDE-Q for the first time among cisgender bisexual plus women and men. Establishing norms for cisgender bisexual plus women and men is important because bisexual plus people are often not delineated from other sexual minority populations (e.g., gay or lesbian), but may have unique health considerations, including eating disorders [1, 31]. Furthermore, most prior community norms of the EDE-Q in women and men did not assess sexual orientation and were presumably predominantly cisgender heterosexual samples [15, 16, 28, 32–36]. A lower percentage of cisgender bisexual plus women reported any occurrence of objective binge episodes, self-induced vomiting, laxative misuse, and excessive exercise than presumed cisgender heterosexual women previously published [16]. Cisgender bisexual plus men had a higher Weight Concern subscale score than presumed cisgender heterosexual men previously published [15].

### Cisgender bisexual plus women

Cisgender bisexual plus women reported the highest scores on the Shape Concern and Weight Concern subscales, consistent with previous EDE-Q norms in presumably cisgender heterosexual young women [16, 28]. The most common disordered eating behaviors reported by cisgender bisexual plus women were dietary restraint (19%) and objective binge episodes (11%). Interestingly, we found lower rates of objective binge episodes, self-induced vomiting, laxative misuse, and excessive exercise in cisgender bisexual plus women compared to presumed cisgender heterosexual women previously published [16]. In a prior study, fasting > 24 h were reported among heterosexual (13%), bisexual (26%), and lesbian (28%) girls [37]. Another study reported binge eating rates in heterosexual (4%), bisexual (10%), and lesbian (7%) women 19–20 years old [38], similar to the rates of binge eating found in this study. However, both studies used heterosexual girls or women as the reference group and thus did not statistically test for differences between bisexual and lesbian girls or women [37, 38]. We did not find significant differences between cisgender bisexual plus women and cisgender lesbian women in EDE-Q Global or subscale scores or disordered eating behaviors.

### Cisgender bisexual plus men

Among cisgender bisexual plus men, we found that the Shape Concern and Weight Concern scores were the highest among all the subscale scores, similar to cisgender bisexual plus women in the present study. These were also the highest subscales previously reported among cisgender gay men [19] and presumed cisgender heterosexual men [15]. Cisgender bisexual plus men in this sample reported higher Weight Concern subscale scores than presumed cisgender heterosexual men previously published [15]. Nearly a quarter of our sample’s cisgender bisexual plus men reported dietary restraint and one tenth reported objective binge episodes in the past 28 days. Laxative misuse and self-induced vomiting were infrequent occurrences. BMI was significantly correlated with the EDE-Q subscales in bisexual plus men, similar to findings previously shown among cisgender gay men [19] and presumed cisgender heterosexual men [15]. We did not find significant differences between cisgender bisexual plus men and cisgender gay men in EDE-Q Global or subscale scores or disordered eating behaviors. These results are consistent with the growing literature demonstrating eating disorder concerns in sexual minority boys and men [31, 37, 39, 40], and add to the literature by providing estimates of eating disorder attitudes and disordered eating behaviors specific to cisgender bisexual plus men.

### Gender differences

There were not significant gender differences in the prevalence of disordered eating behaviors among cisgender bisexual plus women and men. Prior studies among presumed cisgender and heterosexual women [16] and men [15] undergraduate students found that women had higher rates of purging (laxatives and self-induced vomiting) than men [41, 42]. In these same studies, women had higher scores on the EDE-Q Global score and all subscale scores compared to men. In contrast, we did not find significant differences in the EDE-Q Global score or subscale scores among cisgender bisexual plus women and men, except for a higher Eating Concern score among cisgender bisexual plus women compared to men. The lack of gender differences in disordered eating behaviors among cisgender bisexual plus populations highlights some of the unique eating disorder considerations in these populations.

### Minority stress theory

Eating disorder attitudes and disordered eating behaviors among cisgender bisexual plus women and men may in part be explained by the minority stress theory [43], which includes specific biphobic stressors [1]. The minority stress theory posits that prejudice, stigma, and discrimination experienced by sexual minorities, including bisexual plus people, may lead to health disparities. Furthermore, biphobia represents the additional prejudice, stigma, and discrimination experienced by bisexual plus individuals from people with monosexual orientations, including those who identify as gay, lesbian, or heterosexual [1]. Bisexual plus people may face microaggressions, macroaggressions, or bullying from people in the gay and lesbian community due to the assumption that bisexual people may escape some discrimination by heterosexual people by conforming to social expectations of opposite-gender sexual activity and romance [44]. Bisexual plus people may cope with minority stress and biphobia through disordered eating behaviors. The present study results may be consistent with minority stress particularly for cisgender bisexual plus men who reported higher Weight Concern subscales than presumed cisgender heterosexual men.

### Limitations and strengths

There are some limitations to consider in this study. The EDE-Q is based on self-report which may lead to reporting bias. For the purposes of this study, we included cisgender people who identified as bisexual, pansexual, and polysexual, which may obscure possible differences across these groups. We did not include cisgender individuals who identified as “queer.” Some people who identify as queer may see themselves as having a type of bisexual plus identity; others may not. Therefore, we made the a priori decision to exclude these participants; however, identifying eating disorder attitudes and behaviors in people who identify as queer could be an area of future research. Our sample was self-selected, recruited online, highly educated, and mostly white, which limits generalizability as people of color make up a significant portion of the bisexual plus community [45]. Although we were able to assess group comparisons between cisgender lesbian and bisexual plus women, as well as between cisgender gay and bisexual plus men, The PRIDE Study focuses recruitment on sexual and gender minority populations and, thus, we did not recruit cisgender heterosexual women and men for group comparisons. To address this, we identified previously published samples of presumed cisgender heterosexual women [16] and men [15] for comparison; however, these were in young adult university students. Thus, we developed EDE-Q norms for age-matched subsamples of cisgender bisexual plus men and women. Although our age-matched sample of cisgender bisexual plus men was small (*N* = 17), we nonetheless were able to detect a statistically significant difference in the Weight Concern subscale, even after the Bonferroni correction (*p* < 0.005). We did not collect clinical interview data to establish an eating disorder diagnosis and thus were unable to establish clinical cut-offs specific to cisgender bisexual plus women and men, but we did use previously published empirically-derived cutoffs [22]. Despite these limitations, leveraging The PRIDE Study allowed us to describe eating disorder phenomenology in a large number of cisgender bisexual plus people, an understudied sexual minority group.

## Conclusion

We report for the first time EDE-Q norms among cisgender bisexual plus women and men. These findings can help researchers and clinicians to interpret EDE-Q scores in this understudied sexual minority population. Clinicians should be aware that cisgender bisexual plus people may engage in disordered eating behaviors and should consider screening for these behaviors. Future research could examine how factors such as race/ethnicity, age, and socioeconomic status may influence eating disorders and the EDE-Q among cisgender bisexual plus people. In addition, the EDE-Q does not assess for muscularity-oriented concerns and this is an area of future research related to body image in sexual minority populations.

### What is already known on this subject?

Although cisgender bisexual plus women and men may be at elevated risk for eating disorders, community norms of the Eating Disorders Examination Questionnaire (EDE-Q) in these populations are currently lacking.

### What does this study add?

In a large sample of bisexual plus women and men in the US, mean and standard deviation EDE-Q Global score were 1.75 ± 1.26 and 1.56 ± 1.18, while 27.5% and 22.6% scored in the clinically significant range, respectively.
